# Safe and Efficient Intracellular Release of SN38 via Lysosomal-Responsive SN38‑G Loaded Liposomes

**DOI:** 10.1021/acsabm.5c00722

**Published:** 2025-07-08

**Authors:** Pierre-Alain Burnouf, Yu-Cheng Su, Shih-Hung Yang

**Affiliations:** † International Center for Wound Repair and Regeneration, 34882 National Cheng-Kung University, Tainan 704, Taiwan; ‡ Department of Biological Science and Technology, 210861 National Yang Ming Chiao Tung University, Hsinchu 300, Taiwan; § Institute of Biomedical Sciences, 38017 Academia Sinica, Taipei 115, Taiwan

**Keywords:** drug delivery, liposomes, SN38, glucuronide, chemotherapy

## Abstract

Liposomal formulations remain mostly suited for amphiphilic drugs of mild potency. This high selectivity challenges the formulation of potent chemotherapeutics that are preponderantly hydrophobic. Spontaneous accumulation of certain hydrophobic drugs within the lipid bilayer of liposomes leads to aggregation, destabilization, and inadequate drug retention. Here, we depict an alternative approach to load such hydrophobic drugs using SN38, the active form of CPT-11 (Irinotecan, Camptosar), through SN38 glucuronide (SN38-G), its hydrophilic glucuronide prodrug derivative. SN38-G was esterified in acidic methanol or ethanol to produce amphiphilic derivatives SN38-G^met^ and SN38-G^eth^ compatible with liposomal core encapsulation. By employing an internally alkaline pH, core-loaded SN38-G^met^ and SN38-G^eth^ underwent spontaneous hydrolysis, reverting into the hydrophilic SN38-G. This internal conversion resulted in sustained drug retention through lipid-bilayer containment of hydrophilic compounds. Loading above 60% drug-to-phospholipid molar ratio was attained, together with sustained retention over 15 days at 37 °C in simulated body fluids. Our *in vitro* assay demonstrated that SN38-G liposomes were activated to SN38 through a lysosomal beta-glucuronidase-dependent manner, inducing cytotoxicity. This delivery method could be applied to various potent and hydrophobic drugs with challenging delivery requirements.

## Graphical abstract

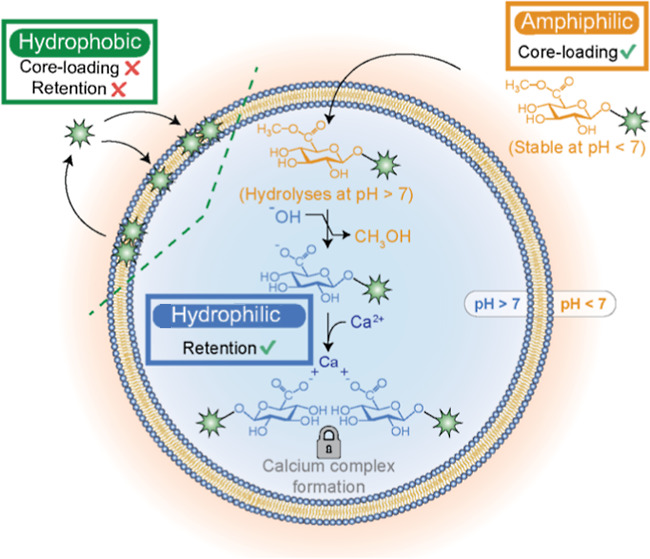

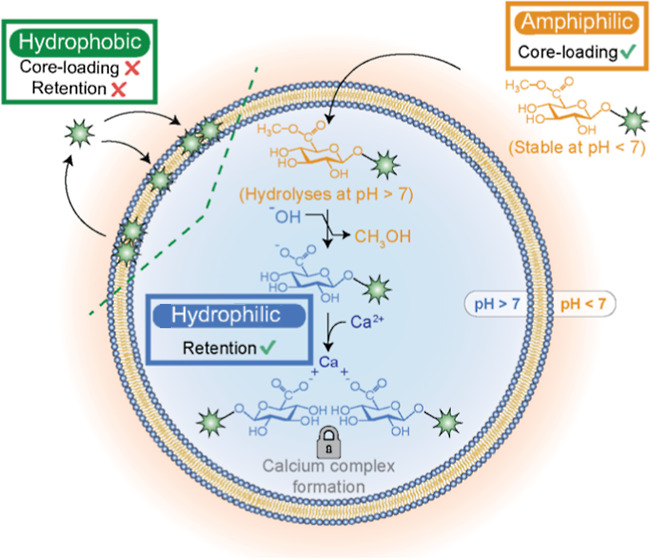

## Introduction

Chemotherapy has safety and efficacy limitations due to its non-specific discrimination between cancerous and healthy tissues.^1,2^ Various drug delivery systems, such as antibody-drug conjugates (ADCs) or nanovehicles like liposomes, have shown promising clinical outcomes by increasing tumor selectivity and reducing interactions with normal cells.^3–5^ Liposomes offer several advantages, including versatility, large cargo capacity, prolonged circulation, and enhanced tumor permeability.^6,7^ Nonetheless, most clinically approved liposomal drugs contain mildly potent amphiphilic chemotherapeutics.^8^ Certain hydrophobic drugs, despite their greater potency and therapeutic potential, are rarely formulated due to inadequate interactions with liposomes, leading to accumulation in the hydrophobic environment of the lipid bilayer, causing aggregation, destabilization, and minimal retention during delivery^9,10^ (Figure 1a).

**Figure 1:**
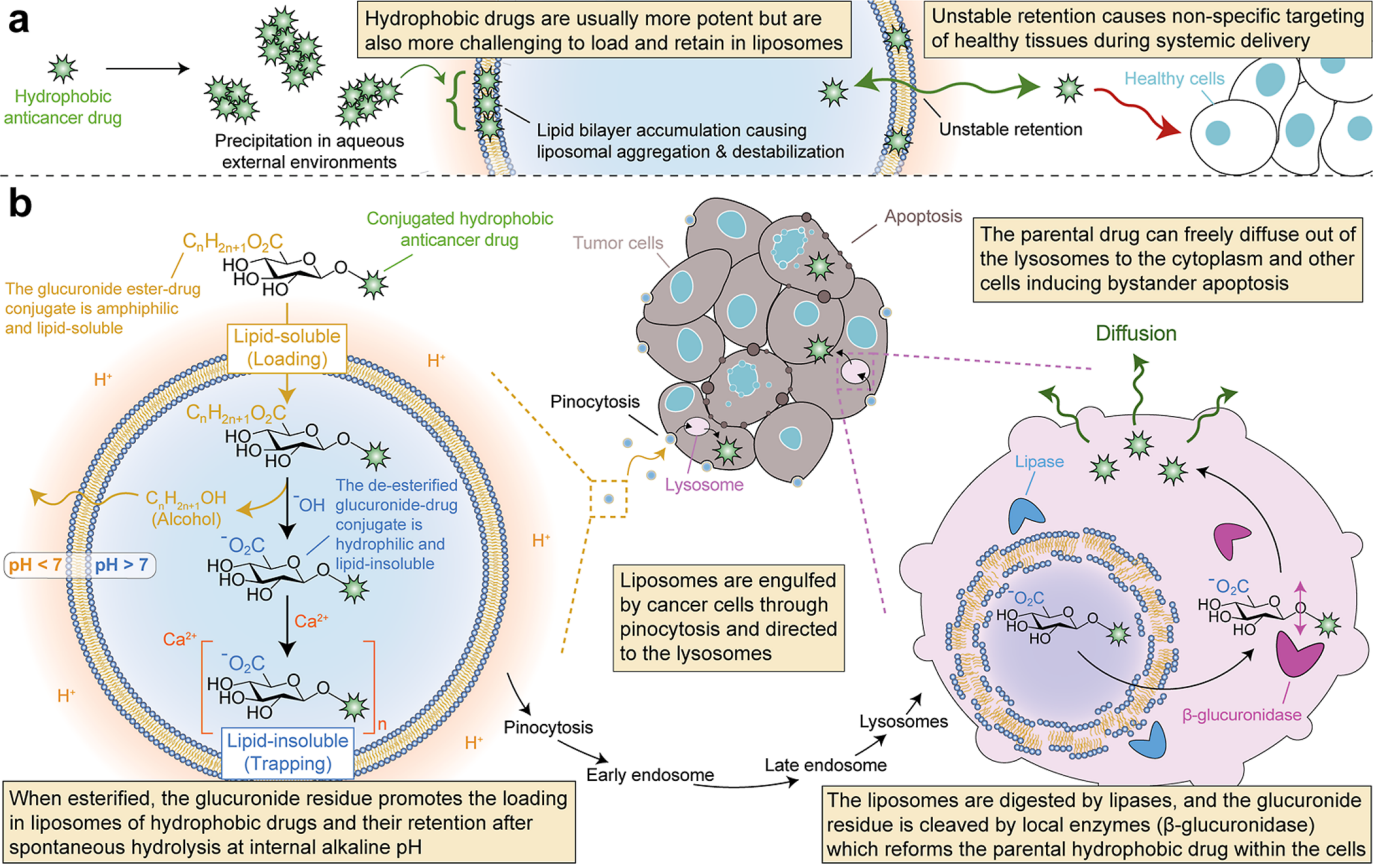
**Applying the glucuronide-drug conjugate liposomes strategy to deliver potent hydrophobic anticancer drugs safely.** (a) Conventionally loaded hydrophobic drugs in liposomes will likely precipitate and accumulate at high concentrations in the liposome’s lipid bilayers, triggering liposome aggregation and destabilization through membrane fusion. During systemic delivery, unstably retained drugs rapidly leak and target surrounding tissues without discrimination, causing unwanted side effects and low treatment efficacy. (b) In the glucuronide-drug conjugate approach, hydrophobic drugs can be reversibly conjugated to a glucuronic acid residue and become hydrophilic. Incubation of hydrophilic glucuronides in acidic alcohol produces amphiphilic ester glucuronides capable of loading in liposomes’ cores at high concentrations. An internal gradient of calcium acetate increases the internal pH, which helps to spontaneously hydrolyze the ester, reforming the hydrophilic glucuronide internally. Internal hydrophilic glucuronides precipitate with free calcium ions for improved retention. Glucuronide-drug conjugates are non-toxic and stably retained during liposomal systemic delivery. They can deliver potent hydrophobic drugs after liposomal internalization by cancer cells and lysosomal enzyme hydrolysis of the glucuronide bond.

This study uses a liposomal glucuronide-drug conjugate to securely deliver such hydrophobic drugs to tumor cells through a lysosomal-responsive release (Figure 1b). The approach involves loading a glucuronide of a hydrophobic drug in liposomes. Through glucuronidation, we may increase the water solubility of a substance by thousands of times,^11–13^ which consequently prevents membrane bilayer crossing. To allow active liposomal loading, we esterified the carboxylic acid group of the glucuronide residue to generate an amphiphilic ester-drug conjugate. This esterification is reversible at elevated pH, allowing the amphiphilic glucuronide ester-drug to reverse to a hydrophilic glucuronide-drug through spontaneous hydrolysis. Accordingly, we produced liposomes containing calcium acetate to form an internally elevated pH and actively loaded the amphiphilic ester-drug. After loading, the ester’s spontaneous hydrolysis resulted in the sustained retention of the hydrophilic glucuronide. In addition, complexation with free calcium ions through the carboxylate group of the glucuronide residue triggered internal precipitation, an essential factor in aiding the retention.^14^ Upon exposure to cancer cells, the liposomes are passively taken up through pinocytosis, which leads to lysosomal fusion.^15,16^ Within the lysosomal compartment, the acidic pH and the presence of lipid-degrading enzymes, such as phospholipases, contribute to the gradual breakdown of the liposomal phospholipid bilayer. This enzymatic degradation compromises the liposomal integrity, allowing encapsulated hydrophilic glucuronide to be released into the lysosomal lumen. The lysosomal enzyme beta-glucuronidase cleaves the glucuronide bond, releasing the potent hydrophobic drug cargo inside the cell.

Thus far, the chemical synthesis of glucuronides for numerous pharmaceutical compounds has been reported^17–21^ and tested preclinically^9,13,18,20–45^ (Supporting Information Table S1), highlighting the potential widespread applicability of the present delivery strategy to various compounds. Here, we employed the glucuronide form (SN38-G) of the potent hydrophobic anticancer compound SN38, which gained approval as an ADC for the treatment of breast cancer^46,47^ and bladder cancer.^48^ It is also the active form of Irinotecan (CPT-11) approved for the treatment of colorectal cancer and small cell lung cancer^49^ as a free form and pancreatic cancer^50^ as a liposomal form.

## Results and Discussion

### Synthesis, Hydrolysis, and Enzymatic Digestion of SN38-G

Accordingly, we formed a loadable amphiphilic SN38-G by esterification in excess alcohol at elevated temperatures using a sulfuric acid (H_2_SO_4_) catalyst. Esterification was performed in either methanol or ethanol, resulting in a methylester (SN38-G^met^) and an ethylester (SN38-G^eth^). Within 12 h, over 95% of SN38-G was esterified to SN38-G^met^ and SN38-G^eth^ (Figure 2a). Both esters are expected to have different levels of amphiphilicity due to their respective ester length, which might influence their loading behavior. We then tested the reversibility of the esterification to regenerate hydrophilic SN38-G through alkaline hydrolysis, which is a crucial step to ensure proper retention after loading. The hydrolysis rates of SN38-G^met^ and SN38-G^eth^ under alkaline conditions at 70 °C, which simulates the elevated pH compartment of liposomes during loading, were adequate. After 1 h, 96.1% (±2.6) of SN38-G^met^ and 95.5% (±2.1) of SN38-G^eth^ were hydrolyzed back to SN38-G (Figure 2b). The hydrolysis duration aligns with the typical incubation time between drugs and liposomes during loading. Lastly, it is crucial to demonstrate that the enzymatic formation of highly potent hydrophobic drug SN38 from SN38-G is feasible. Glucuronides are cleaved by beta-glucuronidase, an enzyme that is active in the lysosomes of mammalian cells. In our assay, SN38-G was rapidly cleaved by beta-glucuronidase at 37 °C to potent hydrophobic drug SN38 (Figure 2c).

**Figure 2:**
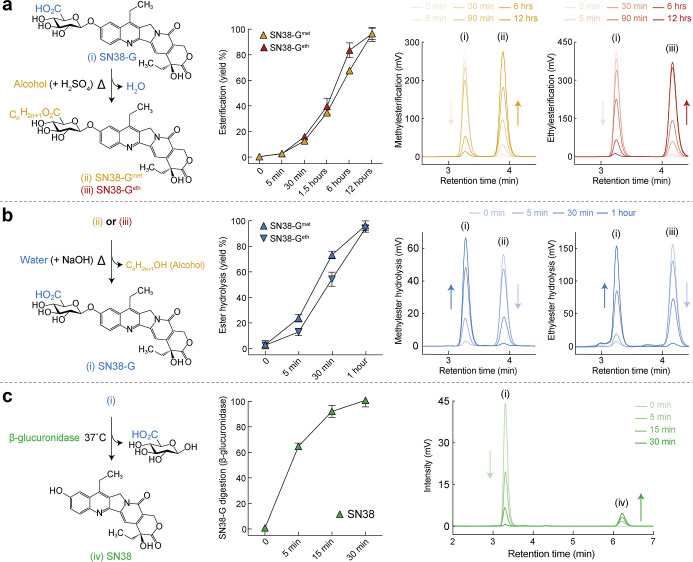
**Esterification along with alkaline hydrolysis of SN38-G and enzymatic conversion to SN38.** (a) Diagram of the formation of esters at glucuronides’ carboxylic acid position. An example is shown for SN38-G esterified in a mixture of alcohol and sulfuric acid (H_2_SO_4_) at elevated temperatures. The nature of the alcohol will determine the nature of the esterification (methylesterification for methanol and ethylesterification for ethanol). Analytical HPLC was performed to monitor the esterification of SN38-G, and results are shown as yields obtained and visual evolution of the chromatograms over 12 h. (b) The reverse reaction was performed in 10 mM HEPES at pH 8. We observed rapid de-esterification of SN38-G^met^ and SN38-G^eth^ to SN38-G through ester hydrolysis and alcohol reformation. (c) The final step involving enzymatic digestion of glucuronide SN38-G to SN38 was achieved by adding enzyme beta-glucuronidase at 37 °C. Error bars: SD, *n* = 3.

HPLC analysis of the sequential formation of intermediates demonstrated near-complete conversion of SN38-G to its esterified derivatives (SN38-G^met^ and SN38-G^eth^), and back to SN38-G, with consistent retention times throughout (Figure 2a,b, Supporting Information Figure S1a,b). These results align with the established chemical principles of esterification and saponification, which predict clean and reversible interconversion under the applied conditions. Subsequent enzymatic digestion using beta-glucuronidase of reformed SN38-G yielded SN38, whose *in vitro* cytotoxicity was comparable to that of a commercial SN38 standard (Supporting Information Figure S1c,d), supporting the structural and functional integrity of both the intermediates and the final product.

Interestingly, elevated levels of beta-glucuronidase in lysosomes of cancer cells compared to those of normal cells have been reported.^51–53^ This phenomenon could enhance the tumor selectivity of glucuronide-drug conjugate liposomes. It is worth noting that in the gut also produces its own beta-glucuronidase, which can pose challenges during CPT-11 chemotherapy.^54,55^ Upon administration, CPT-11 is converted to SN38 in the liver and rapidly glucuronidated to SN38-G. SN38-G is excreted in the bile and released into the small intestine. In the intestinal lumen, beta-glucuronidase from gut microbiota enzymatically hydrolyzes SN38-G to SN38. While this mechanism may increase local drug activation against colorectal cancer, it is also associated with severe gastrointestinal side effects like diarrhea, nausea, abdominal pain, and loss of appetite.^56^ It is expected that liposomal SN38-G administration alleviates these unwanted effects as liposomes are not typically excreted through the bile into the intestinal lumen, a pathway commonly associated with endogenous substances or drugs undergoing hepatobiliary elimination.

### Water Solubility and Partitioning (Log *P*)

Water solubility and partitioning (log *P*) are important factors for determining the drugs’ compatibility with loading and retention in liposomes (Figure 3d). A drug with low water solubility (high log *P*) may precipitate in the liposome’s external environment or accumulate in the lipid bilayer, causing destabilization.^9,10^ Conversely, a drug with high water solubility and negative log *P* is unlikely to cross the lipid bilayer, making it unsuitable for active drug loading. However, such a drug would exhibit strong retention if it can be loaded. Amphiphilic compounds are ideal for loading, because they can be solubilized at high concentrations in aqueous environments without precipitation. They also tend to have a moderate affinity with lipid bilayers, facilitating efficient crossing without excessive accumulation.

**Figure 3:**
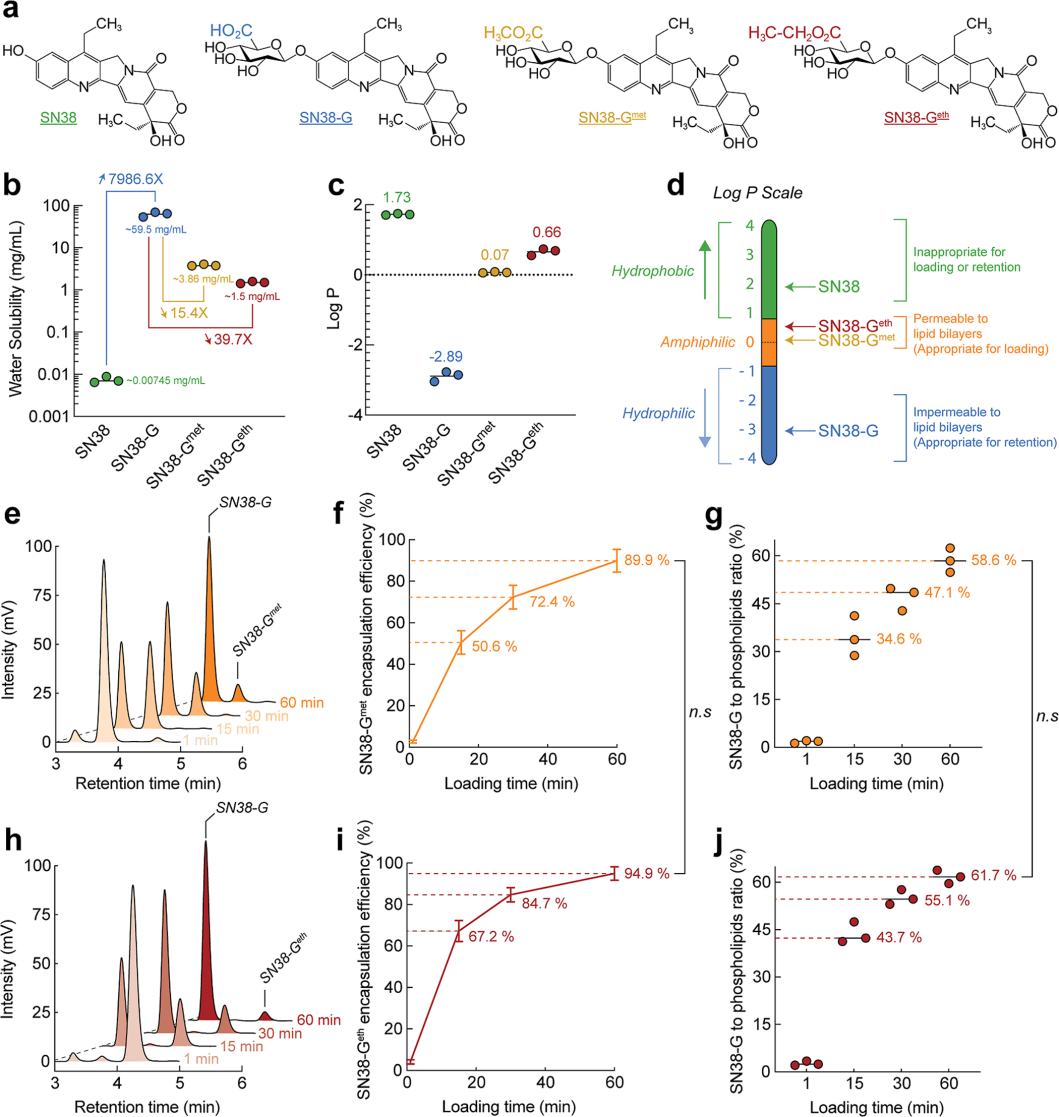
**Comparison of the water solubility, partitioning, and loading capabilities in liposomes.** (a) Molecular structures of the parental drug (SN38), the glucuronide form (SN38-G), the methylesterified glucuronide (SN38-G^met^), and ethylesterified glucuronide (SN38-G^eth^) assessed for (b) water solubility and (c) 1-octanol-to-water partitioning assay (Log *P*). (d) A diagram of the Log *P* scale where amphiphilic, hydrophobic, and hydrophilic zones are delimited with relation to liposomal drug loading and retention. The time-dependent drug loading chromatograms (e,h), encapsulation efficiencies (f,i), and final SN38-G-to-phospholipids ratios (g,j) in liposomes were monitored by a combination of analytical HPLC and phospholipid Bartlett’s assay for SN38-G^met^ (e–g) and SN38-G^eth^ (h–j). Error bars: SD, *n* = 3. Statistical significance of differences in mean values: n.s not significant.

Water solubility tests were conducted in a pH 6 buffer (Figure 3b) to prevent the hydrolysis of the ester bond. As expected, the solubility of SN38 was low at 7.45 μg/mL (±0.98). Adding a glucuronide residue to SN38 (SN38-G) dramatically increased solubility to 62.8 mg/mL (±8.72), an approximately 8500-fold gain. Methylesterification of SN38-G to SN38-G^met^ decreased water solubility of SN38-G by 16-fold to 3.86 mg/mL (±0.13), while ethylesterification to SN38-G^eth^ further reduced it to 1.5 mg/mL (±0.87), nearly 42-fold decrease.

The log P, defined here by partitioning between 1-octanol and pH 6 buffer (Figure 3c), was measured to determine compound affinity for organic phases such as the lipid bilayer. SN38 had a Log *P* of 1.725 (±0.02), indicating a 53-fold higher affinity for the organic phase. As a glucuronide-drug conjugate, SN38-G exhibited significantly increased water solubility and reversed affinities toward the aqueous phase. SN38-G had a Log *P* of -2.89 (±0.12), showing over 775-fold higher affinities for the aqueous phase. While strong water affinities are favorable for liposomal retention, they can hinder encapsulation efficiencies. Therefore, transient esterification in methanol or ethanol is essential to achieve more suitable Log *P* values for active drug loading. SN38-G^met^ had a Log *P* of 0.069 (±0.013), while SN38-G^eth^ had a Log *P* of 0.66 (±0.11), indicating their amphiphilic nature and potential aptitude for efficient drug loading. Similar distribution trends were observed using soybean oil as the organic phase (Supporting Information Figure S2); however, the glucuronide derivatives showed slightly reduced partitioning into the oil phase compared to octanol. This may reflect differences in solvent polarity and the influence of the polar glucuronide moiety.

### Loading Potential and Stability in Simulated Physiological Fluids

Liposomes were formed with an aqueous core of 250 mM calcium acetate , and dispersed in 10 mM MES and 240 mM sodium sulfate (adjusted to pH 6) using size-exclusion chromatography (Sephadex G50). Before loading, methanol, and ethanol solvents were removed from drug stock solutions through evaporation at 95 °C for 45 min, leaving SN38-G^met^ and SN38-G^eth^ in DMSO. The drugs were then added to the liposomes at a 65% drug-to-phospholipids molar ratio at 70 °C and incubated for 60 min. Loading progress was monitored using HPLC after harvesting and lysing the samples in 70% methanol and 25 mM citric acid (pH 3.0) at 70 °C for 10 min. Significant increases in SN38-G amounts were observed over time, confirming loading and internal hydrolysis at elevated pH (Figure 3e,h). The kinetics of loading and conversion between SN38-G^met^ and SN38-G^eth^ surprisingly showed no significant difference, with average values of 89.9% (±5.6) and 94.9% (±3.3) of the initial amounts, respectively (Figure 3f,i). The final drug-to-phospholipids ratio was 58.6% (±3.9) for SN38-G^met^ and 61.7% (±2.1) for SN38-G^eth^ (Figure 3g,j).

The localized hydrolysis of SN38-G^met^ and SN38-G^eth^ within the aqueous core of calcium-acetate-loaded liposomes raises an important question regarding the potential susceptibility of liposomal phospholipids to similar degradation. However, this concern is mitigated by the distinct chemical environments in which these reactions occur. The esterified SN38-G derivatives reside in the aqueous lumen, where a basic pH is generated by a calcium acetate gradient, promoting the hydrolysis of the labile glucuronide ester bonds. In contrast, the ester linkages of phospholipids are embedded within the hydrophobic bilayer, where water activity and hydroxide ion concentration are minimal, making base-catalyzed hydrolysis under these conditions unlikely. Furthermore, the hydrolysis of phospholipid esters typically requires harsh alkaline conditions or enzymatic catalysis, neither of which are present in our formulation. This spatial and chemical separation ensures that only the drug conjugates are hydrolyzed, while the lipid membrane remains intact. Previous studies have shown that phospholipid bilayers remain stable during remote loading with calcium acetate gradients.^57,58^ In our study, the preserved morphology of the liposomes post-loading, as confirmed by cryo-EM imaging (Figure 4a), further supports the structural integrity of the bilayer under these conditions. In addition, both SN38-G^met^ and SN38-G^eth^ formulations showed nonsignificant changes in size and PDI compared to empty liposomes only containing calcium acetate (Supporting Information Figure S3). These results indicate that drug loading did not substantially alter the physical properties of the liposomes.

**Figure 4:**
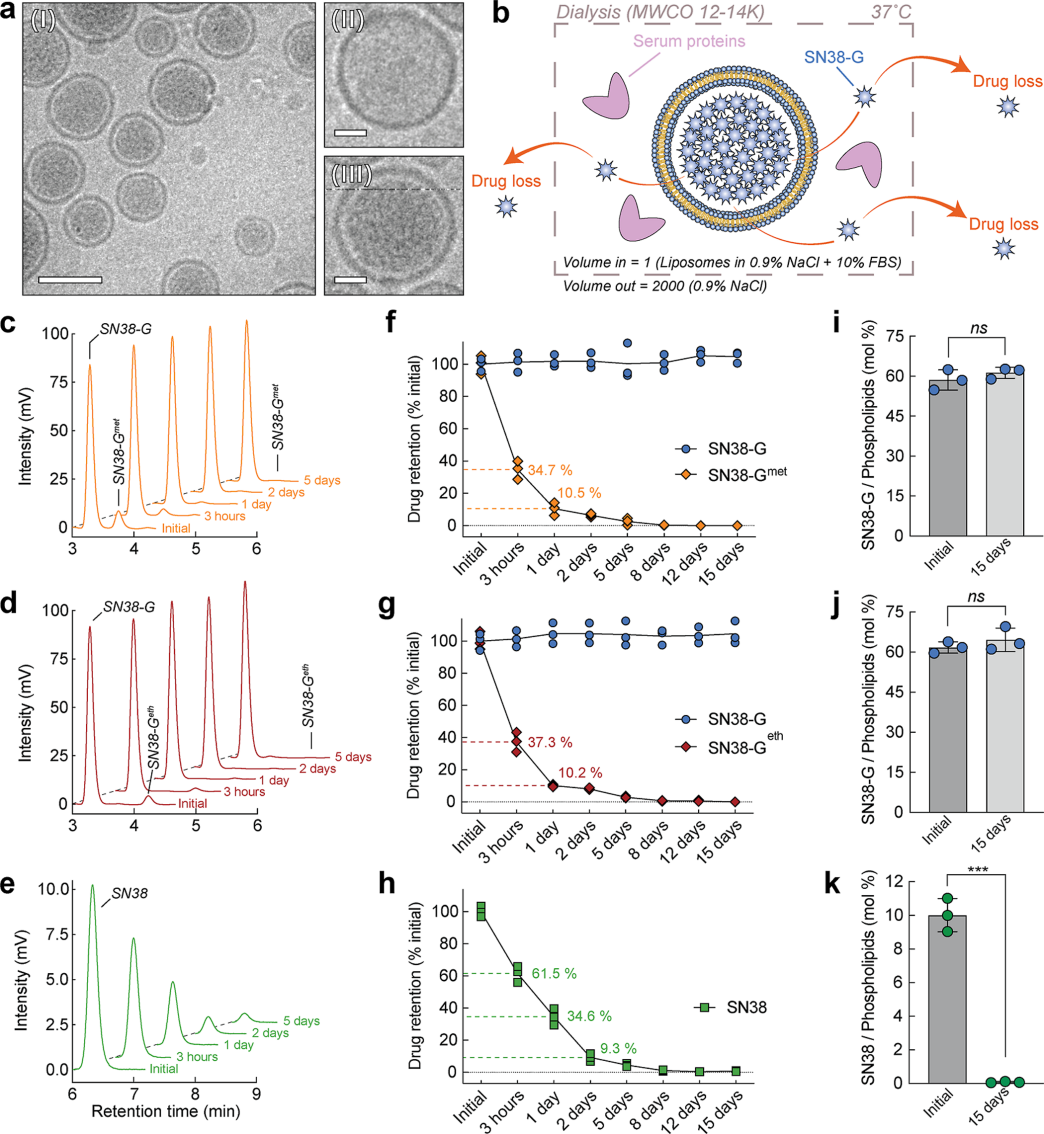
**Cryogenic electron microscopy visualization and stability in simulated physiological fluids.** (a) Wide-field cryogenic electron micrograph of SN38-G liposomes (I, scale = 100 nm) and zoomed-in views of an empty (calcium acetate) liposome (II, scale = 20 nm) compared to a loaded liposome (III, scale = 20 nm). (b) Schematic representation of the stability assay assessing the retention of drugs inside liposomes. HPLC analysis of the liposomal content, drug retention, and final drug-to-phospholipid molar ratio for the loading of SN38-G^met^ (c,f,i), SN38-G^eth^ (d,g,j), and SN38 (e,h,k). Error bars: SD, *n* = 3. Statistical significance of differences in mean values: n.s not significant, ***p < 0.001.

To address the safety profile of the ethylester (SN38-G^eth^) and methylester (SN38-G^met^) derivatives, we considered the potential impact of residual alcohols generated during hydrolysis within the liposomal core. Upon encapsulation, both ester prodrugs are hydrolyzed under basic conditions to regenerate SN38-G, releasing methanol or ethanol as a byproduct. These small, uncharged alcohols are known to readily permeate lipid bilayers. Notably, ethanol has been shown to diffuse more rapidly across phospholipid membranes than methanol,^59^ attributed to its slightly higher hydrophobicity and partitioning ability. This difference suggests that ethanol is more efficiently cleared from the liposomal interior following hydrolysis, favoring the use of SN38-G^eth^ from both a safety and formulation standpoint. To minimize the presence of residual alcohols, our formulation protocol includes a post-loading dialysis step, which facilitates the removal of free methanol or ethanol from the liposomes into the surrounding medium. Although we did not directly quantify alcohol levels in the dialysate, the physicochemical properties of methanol and ethanol support the expectation that they are effectively eliminated during this step. From a toxicological perspective, the theoretical amount of alcohol generated is minimal. Complete hydrolysis of 1 mg of SN38-G^met^ or SN38-G^eth^ would yield approximately 1.72 μmol (55 μg) of methanol or 1.68 μmol (77 μg) of ethanol, respectively. While the low quantities involved suggest a negligible systemic impact, it is worth noting that methanol is inherently more toxic than ethanol. This further supports the selection of SN38-G^eth^ for future development, as it offers improved safety due to both its metabolic byproduct and its more efficient clearance from liposomes. These considerations collectively highlight the pharmacological and formulation advantages of using ethylester derivatives for future developments.

Internally generated SN38-G can form complexes with internal calcium ions, forming stable aggregates. The calcium acetate gradient-based loading strategy, developed by Clerc and Barenholz (US Patent No. 5,939,096), utilizes calcium-induced intraliposomal precipitation to enhance the stability of liposome-encapsulated drugs. This approach was originally developed and extensively validated for amphiphilic weak acids that are on their own capable of diffusing across the liposomal membrane.^60,61^ Improved drug retention in this system is attributed to the formation of stable intraliposomal complexes between calcium ions and weak acid drugs, effectively minimizing leakage. However, a key limitation of the method is its reliance on the drug’s intrinsic amphiphilicity. In contrast, our formulation strategy expands the applicability of calcium acetate-based liposomes to non-amphiphilic weak acids and hydrophobic drugs, which are typically excluded from such loading techniques.

Based on our loading calculations, each individual liposome with an average diameter of 100 nm contains approximately 48,000 SN38-G molecules, complexing with about 61.5% of the free calcium ions available. We speculate that maximum loading capacity here is not limited by the amount of calcium counterions but rather capped by spatial saturation. To illustrate, Doxil liposomes have ample empty space due to the rod-like structure of doxorubicin-sulfate complexes, containing only 10,000–15,000 doxorubicin molecules per liposome.^62^ In contrast, drug-forming space-filling complexes like CPT-11, which share a similar structural scaffold with SN38-G, achieved a drug-to-phospholipid molar ratio of approximately 84.6% (Onivyde). (Source: FDA prescribing information).

Internal complex formation has been shown to enhance the sustained retention of drugs within liposomes.^14^ Cryo-EM imaging (Figure 4a­(I)) of SN38-G-loaded liposomes revealed visible complex formation with calcium, that saturated the entire core space compared to non-loaded liposomes (Figure 4a­(II,III)). Dialysis assay (Figure 4b) in 0.9% NaCl with 10% FBS at 37 °C showed rapid clearance of residual amphiphilic SN38-G^met^ (Figure 4c,f) and SN38-G^eth^ (Figure 4d,g) from liposomes, confirming the efficient removal of unconverted ester prodrugs. In contrast, generated SN38-G exhibited excellent retention within the liposomes, maintaining a stable drug-to-phospholipid molar ratio over 15 days (Figure 4i,j). This stability is consistent with the physicochemical properties of SN38-G and its intraliposomal complexation with calcium, which limits membrane permeability.

Comparative loading of parental drug SN38 in liposomes was tested. SN38, being neutral in the liposomal external acidic pH, readily interacts with lipid bilayers, potentially reaching the internal compartment. The elevated internal pH triggers the lactone ring opening of SN38 to give a carboxylate, which can be complexed with internal calcium ions to aid retention. DMSO was added at a final concentration of 20% to facilitate the drug loading as previously demonstrated.^63^ Post-loading, non-encapsulated SN38 was removed using size-exclusion chromatography. Despite the addition of DMSO, SN38 loaded at a modest 10.01% (±0.99) drug-to-phospholipids molar ratio. Furthermore, SN38 liposomes exhibited weak retention, with over 99.7% content loss over 15 days (Figure 4e,h,k).

Previously, liposomal formulations of SN38 were prepared using a thin-film hydration method, a passive drug-loading strategy.^64^ This method involved dissolving lipids and SN38 in ethanol, forming a dried lipid/SN38 film and rehydrating it with an aqueous solution to form the liposomes. Plasma clearance studies in BALB/c mice showed no difference between SN38-loaded liposomes and free SN38 groups, suggesting rapid drug release from the liposomes. Similar results were observed with NeoPharm, Inc.’s liposomal SN38 (LE-SN38) in a phase-2 clinical trial for metastatic colorectal cancer.^65,66^ Unfortunately, the treatment did not yield the desired tumor response, likely due to the limitations of the passive thin-film hydration method, which lacks an effective retention mechanism. We believe that, in these cases, SN38 likely experienced burst release from liposomes upon dilution, resembling the behavior of a free SN38 administration. In contrast, in our study, the loading of a glucuronide-modified SN38 (SN38-G) demonstrated remarkable liposomal stability in simulated physiological fluids.

In recent years, increasing efforts have been devoted to the development of SN38-based nanoparticle formulations to overcome the drug’s poor aqueous solubility, limited stability, and lack of suitable delivery strategies.^67–70^ These approaches include prodrug designs, covalent lipid conjugates, and polymeric nanocarriers, all aiming to improve SN38’s pharmacokinetic profile and enhance its antitumor activity. This growing interest highlights the ongoing challenge of translating SN38 into an effective clinical agent and underscores the potential value of stable release-controlled liposomal systems such as SN38-G liposomes in addressing this unmet need.

### Cell Cytotoxicity and Intracellular Regeneration of Parental Drug SN38

We then compared the *in vitro* cytotoxicity of SN38-G liposomes, free SN38-G, and free SN38 against MCF-7 and MDA-MB-231 breast adenocarcinoma cells (Figure 5a,b). As expected, free SN38, our positive control, showed the highest anticancer activity, with IC_50_ values of 5.82 (MCF-7) and 24 nM (MDA-MB-231). The addition of a glucuronic acid moiety to SN38 (free SN38-G) significantly reduced its toxicity by 728.5-fold (MCF-7) and 550.5-fold (MDA-MB-231). Encapsulation of SN38-G in liposomes resulted in cytotoxicity comparable to that of free SN38-G, with IC_50_ values of 0.918 μM (MCF-7) and 12.17 μM (MDA-MB-231). This result contrasts with other liposomal drugs that often exhibit significantly lower efficacy than their free drug counterparts.^71^ A Comparative assay showed similar IC_50_ values for free SN38 and SN38-loaded liposomes (Supporting Information Figure S4), likely due to rapid drug release from the liposomes. In contrast, CPT-11 exhibited low cytotoxicity in MCF-7 cells, as previously reported,^72^ highlighting the potential utility of SN38-G liposomes in models with limited CPT-11 sensitivity.

**Figure 5:**
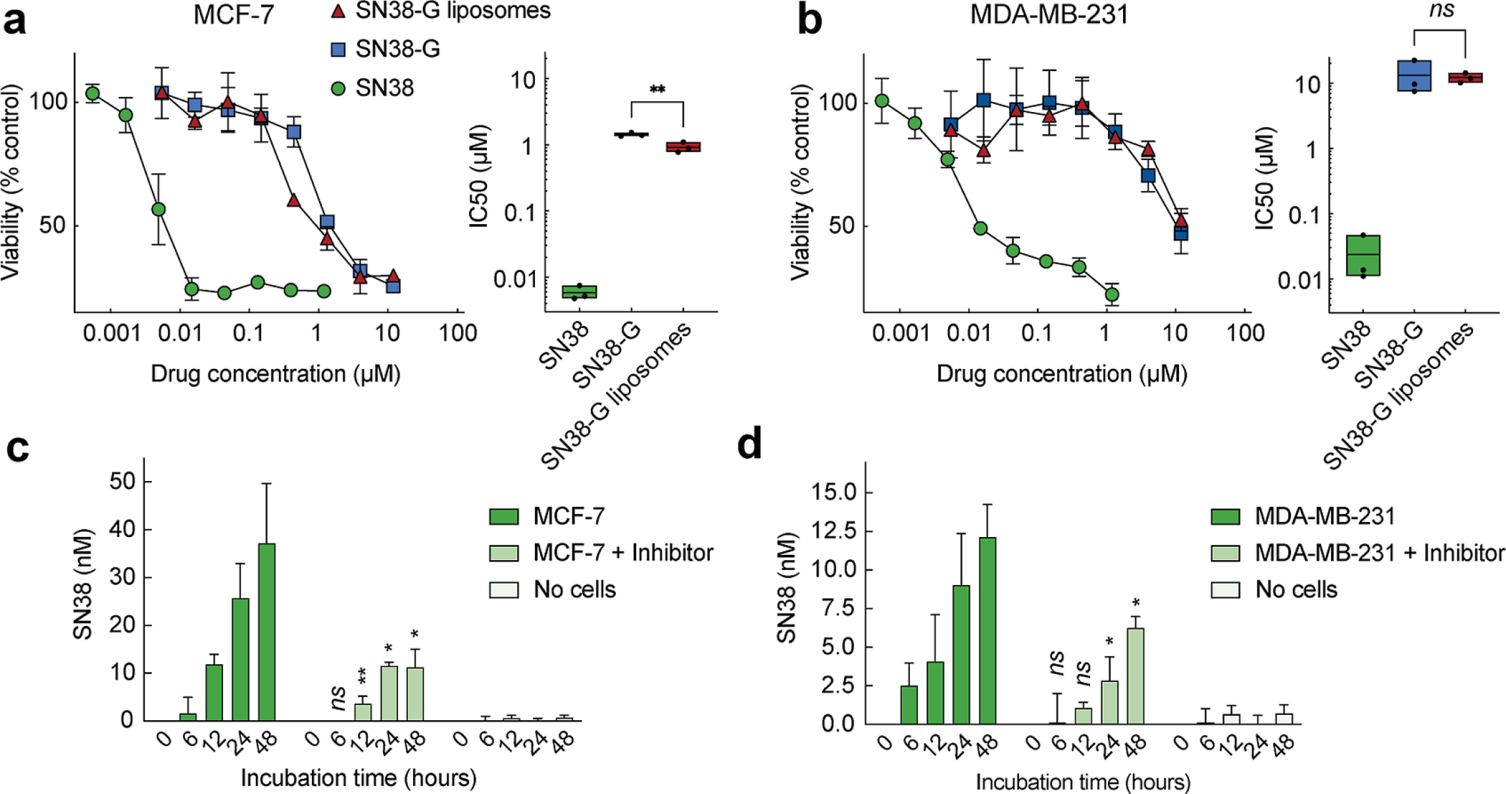
**Cell cytotoxicity and intracellular regeneration of parental drug SN38.** Dose-dependent cytotoxicity of breast adenocarcinoma cells MCF-7 (a) and MDA-MB-231 (b) against SN38-G liposomes, free SN38-G, and parental drug SN38. *In vitro* regeneration of parental drug SN38 from MCF-7 (c) and MDA-MB-231 (d) exposed to SN38-G liposomes with or without beta-glucuronidase inhibitor, d-saccharic acid 1,4-lactone. Error bars: SD, *n* = 3. Statistical significance of differences in mean values: n.s., not significant, *p < 0.1, **p < 0.01.

Notably, while the liposomes exhibited potent cytotoxicity in MCF-7 cells, their toxicity toward non-cancerous NIH/3T3 fibroblasts was markedly lower at equivalent concentrations (Supporting Information Figure S5), indicating a level of selectivity for cancer cells and supporting the formulation’s preliminary biocompatibility. Empty liposomes exhibited no detectable cytotoxicity in both MCF-7 and 3T3 cells, confirming the biocompatibility of the carrier system (Supporting Information Figure S6).

Liposomal drugs often exhibit reduced potency *in vitro* compared with free drugs, which can more readily diffuse across cellular membranes and reach intracellular targets. However, the *in vivo* advantages of liposomal formulations, such as prolonged circulation, passive accumulation in tumor tissue via the enhanced permeability and retention (EPR) effect, and reduced systemic toxicity, play a pivotal role in their therapeutic success. This contrast is particularly important in our case as the free form of SN38 is highly toxic and unsuitable for systemic administration, while the prodrug SN38-G, though less toxic, is rapidly cleared from circulation. Encapsulation within liposomes helps to overcome both challenges by improving both the tolerability and pharmacokinetic stability.

Noticeably, the sensitivity difference to SN38-G liposomes between MCF-7 and MDA-MB-231 was over 10-fold. We investigated whether this discrepancy correlated with the level of conversion to parental SN38 (Figure 5c,d). To assess this, we incubated MCF-7 and MDA-MB-231 cells with 2 μM SN38-G liposomes and measured the conversion to SN38. Our control experiment without cells demonstrated no cell-independent degradation or hydrolysis of the liposomes to SN38. However, MCF-7 produced nearly 2.5-fold higher levels of SN38 from an equal concentration of the SN38-G liposome.

We also investigated whether the SN38 production by cells was dependent on the lysosomal enzyme beta-glucuronidase by adding 100 μM of a competitive inhibitor, d-saccharic acid 1,4-lactone. The inhibitor effectively suppressed the production of SN38 in cells, to a significant extent. This observation suggests that liposomes undergo targeted degradation in the lysosomes, thereby exposing SN38-G to beta-glucuronidase-dependent hydrolysis, leading to intracellular production of SN38.

Due to the insufficient intrinsic fluorescence of SN38 and its derivatives, direct visualization by confocal microscopy was not feasible. As an alternative approach, we previously developed a fluorescent model system using liposomal fluorescein di-glucuronide,^9^ which serves as a proxy for glucuronide-modified drugs. In that study, fluorescein di-glucuronide, a non-fluorescent compound, was loaded into DiD-labeled liposomes via its methylester form using the same calcium acetate gradient method described here. Upon cellular uptake, the liposomes accumulated in lysosomes, where enzymatic cleavage by beta-glucuronidase released fluorescent fluorescein, enabling us to visualize lysosomal activation and cytoplasmic diffusion. These observations support a mechanism similar to that of lysosomal uptake and enzymatic activation for SN38-G in the current system.

## Conclusion

In summary, we developed a lysosomal-responsive intracellular release of SN38 by encapsulating its glucuronide derivative, SN38-G in liposomes. Derivation to glucuronides presents several advantages, including (1) fine-tuning the physicochemical properties of drugs from amphiphilic to hydrophilic, facilitating the efficient loading and stable retention within liposomes; (2) mitigating the risks associated with chemotherapy by encapsulating low-toxicity glucuronide compounds, thereby reducing potential side effects; (3) facilitating intracellular delivery and selectivity of potent drugs to cancer cells through their enhanced lysosomal beta-glucuronidase activity. This proof-of-principle study represents a significant advancement in the design and formulation of potent hydrophobic drugs within liposomes, with the aim to enhance the therapeutic efficacy of promising compounds through novel approaches.

## Methods

### Chemicals

Distearoylphosphatidylcholine (DSPC) and 1,2-distearoyl-*sn*-glycero-3-phosphoethanolamine-*N*-[methoxy­(polyethylene glycol)-2000] (DSPE-PEG) were purchased from Avanti Polar Lipids (Alabaster, AL, USA). Ultrapure cholesterol (>99%), calcium acetate monohydrate, sodium sulfate, 2-(*N*-morpholino)­ethanesulfonic acid (MES), Cell Counting Kit-8 (CCK-8), d-saccharic acid 1,4-lactone, Fiske&Subbarow reducer, and ammonium molybdate were purchased from Sigma-Aldrich (St. Louis, MO, USA). SN38, SN38 glucuronide (SN38-G), and CPT-11 were purchased from TLC Pharmaceutical Standards (Ontario, Canada).

### Cell Lines

Breast adenocarcinoma cells MCF-7 and MDA-MB-231 were kindly provided by Dr. Jyun-Yuan Huang (Ducolege Biotechnology CO., LTD). NIH/3T3 fibroblasts were obtained from the American Type Culture Collection (ATCC, Manassas, VA, USA). All cells were cultured in Dulbecco’s Modified Eagle Medium (DMEM) supplemented with 5% heat-inactivated FBS, 3.7 g/L sodium bicarbonate, 100 units/mL penicillin, and 100 μg/mL streptomycin in a 5% CO2 humidified atmosphere at 37 °C. All cells were tested for mycoplasma and propagated for less than 6 months.

### Synthesis of Amphiphilic SN38-G^met^ and SN38-G^eth^ from SN38-G

Esterification was performed by dissolving SN38-G in methanol or ethanol with an acid catalyst, sulfuric acid (H_2_SO_4_). DMSO was added to help solubility during ester formation, as precipitation might occur, particularly for ethylesterification. Regarding methylesterification, as the reaction is relatively more efficient, we diluted pure H_2_SO_4_ 10-fold with methanol before use. Typically, each batch of methylesterified SN38-G (SN38-G^met^) was prepared using 10 mg of SN38-G in 200 μL of DMSO, 1.5 μL of diluted H_2_SO_4_, and 798.5 μL of pure methanol, achieving a 1 to 0.16 molar ratio between SN38-G and H_2_SO_4_ and maintaining methanol in excess. Ethylesterification was performed similarly by the addition of 10 mg of SN38-G to 200 μL of DMSO, 1.5 μL of pure H_2_SO_4_, and 798.5 μL of pure ethanol to achieve a molar ratio of 1 to 1.6 between SN38-G and H_2_SO_4_, and keeping ethanol in excess. In both conditions, the temperature was increased to 65–75 °C, and respective ester formations were monitored by analytical HPLC by diluting 2 μL of the reaction mixture in 248 μL of HPLC mobile phase and injecting 2.5 μL with the detector set to an excitation/emission wavelength of 375/430 nm.

### Hydrolysis of Amphiphilic SN38-G^met^ and SN38-G^eth^ to SN38-G

Hydrolysis of SN38-G^met^ and SN38-G^eth^ was performed in 10 mM HEPES adjusted to pH 8 to simulate the alkaline internal compartment of the liposomes during drug loading. We monitored the reaction at 70 °C and took samples at 0, 5, 30, and 60 min of incubation time. Each sample was diluted in HPLC mobile phase (70% methanol, 25 mM citric acid, pH 3) and incubated at 70 °C 10 min before injecting in the HPLC to ensure the reformation of the lactone ring.

### Water Solubility and Log *P*

We investigated the water solubility and Log *P* (partitioning between water and 1-octanol) under closed lactone ring formation. We added SN38, SN38-G, SN38-G^met^, and SN38-G^eth^ in 10 mM MES pH 6 until it reached saturation and stopped dissolving. Saturated solutions were kept under agitation at room temperature for 24 h and centrifuged at 20,000*g* for 30 min at 24 °C. Analytical HPLC was then used to quantify the supernatants. The partitioning between 1-octanol and water was determined in 500 μL of 10 mM MES at pH 6 and 500 μL of 1-octanol. A volume of 20 μL of supernatants from the water solubility assay was added to the water phase to ensure that the compounds were below the water saturation threshold. We allowed equilibration between the 2 phases for 24 h at room temperature under constant shaking. After this, the tubes were centrifuged at 20,000*g* for 30 min at 24 °C, and samples from each phase were analyzed by analytical HPLC. Using 70% methanol mobile phase conveniently dissolves 1-octanol, ensuring precise estimation of drugs’ quantities. The final Log *P* (partition coefficient) was determined by the Log of the fraction of the amount of drug found in the 1-octanol phase over the amount of drug found in the water phase as follows

1 $$\mathrm{Log}Pvalue=\mathrm{Log}\left({\left[drug\right]}_{octanol}{/\left[drug\right]}_{water}\right)$$

### Formation of the Liposomes

Powdered DSPC, DSPE-PEG, and cholesterol were dissolved in ethanol right before use. Warming the solutions above 37 °C helps to ensure complete solubility of the lipids. The liposomes were formulated by mixing DSPC, DSPE-PEG, and cholesterol at molar ratios of 59.25%, 0.75%, and 40%, respectively, in a borosilicate glass flask. We evaporated the ethanol under a vacuum on a rotary evaporator (Buchi Rotavapor R-100) at 45 °C. When most of the ethanol appears to be removed, the temperature is raised to 65 °C and maintained under vacuum for another 20 min. The lipid film was rehydrated with a solution of 250 mM calcium acetate to a final lipid concentration of 15 mg/mL. The liposomal suspension was then extruded 11 times at 65 °C through a 100 nm polycarbonate membrane using an Avanti mini extruder. The external solution of calcium acetate was replaced by a poly­(ether sulfone) membrane (0.22 μm)-filtered solution of MES (10 mM) and sodium sulfate (240 mM) with pH adjusted to 6 with the addition of NaOH on a Sephadex G50 size-exclusion column, using 20-fold gel volume per liposome volume. Collected liposomes were stored in the dark at 4 °C for a maximum of 2 weeks before use. The final lipid concentration was determined by a modified Bartlett’s phosphorus assay.

### Phospholipids Assay

We based our assay on Bartlett’s protocol. Phosphate standard (0, 25, 50, 100, and 125 μL of 0.65 mM KH_2_PO_4_) and sample (typically 5, 15, and 30 μL of liposomes) were added in triplicate to glass tubes, followed by the addition of 400 μL of 72% perchloric acid. We mixed the tubes through vortex and heated to 180 °C for 30 min, cooled down the tubes at room temperature and added 1 mL of double-distilled water, 200 μL 5% (w/w) ammonium molybdate solution, and 50 μL of Fiske&Subbarow reducer (0.16 g of 4-amino-2-naphthyl-4-sulfonic acid reagent in 1 mL of double-distilled water), and mixed the tubes and heated at around 100 °C for 10 min before reading the absorbances at 830 nm.

### Drug Loading

We loaded SN38-G^met^ or SN38-G^eth^ at a starting drug-to-phospholipid (D/pL) molar ratio of 65%. Methanol or ethanol solvents from stock solutions were removed by drying at 90 °C for 45 min, only leaving SN38-G^met^ or SN38-G^eth^ in DMSO. Typically, liposomes (2 μmol) and SN38-G^met^ or SN38-G^eth^ (1.3 μmol) were mixed in a total volume of 500 μL complemented with liposomal external medium (10 mM MES, 240 mM sodium sulfate, pH 6). We preheated the liposomes and the drugs separately to 70 °C before mixing them and maintained the temperature at 70 °C for 1 h in a water bath with occasional gentle swirling. We generally took samples for analytical HPLC at initial mixing (∼1 min) and after 15, 30, and 60 min to follow the encapsulation efficacy and D/pL ratio. After 1 h of loading, we transferred the tubes on ice for at least 10 min and removed non-encapsulated drugs by dialysis at 4 °C in a 1–250 volume ratio of 17 mM HEPES and 144 mM NaCl, exchanged 3 times every 4–6 h. Liposomal SN38-G was stored at 4 °C in the dark before use.

### Cryo-Transmission Electron Microscopy

Empty liposomes or SN38-G liposomes were used for imaging at 2 mg/mL of phospholipids. Images were collected on a JEM-2100F transmission electron microscope at 10K and 20K magnification and processed through ImageJ software.

### Drug Stability

We diluted SN38-G liposomes at 1 mg/mL of phospholipids in 2 mL of 0.9% NaCl, complemented with 10% FBS, before transferring to dialysis membrane (Visking Tubing, diameter 15.9 mm, molecular weight 12,000–14,000). We ran the dialysis at 37 °C in the dark against 2 L, to which we added 0.02% sodium azide to prevent bacterial growth, under constant stirring. Samples of 2 μL of liposomes were taken at different intervals and diluted in 248 μL of HPLC mobile phase (70% methanol, 25 mL citric acid, pH 3), lysed for 10 min at 70 °C, and injected (Volume = 2.5 μL) in the C18 reverse phase HPLC system to analyze the total remaining amount of encapsulated SN38-G.

### Analytical HPLC

Our HPLC analyses were performed on a Hitachi 5110 pump combined with a Hitachi 5440 fluorescence detector and C18 column (SCpak ODS-P SpectroChrom, 250 mm × 4.6 mm ID, 5 μm). The mobile phase comprised 70% methanol and 25 mM citric acid, pH 3, used at a 1 mL/min flow rate. SN38-G, SN38-G^met^, and SN38-G^eth^ were analyzed at 375/430 nm (ex/em), and SN38 was analyzed at 375/540 nm (ex/em). Liposome samples were incubated at 70 °C for 5 min in the HPLC mobile phase before injection to lyse the liposomes and reform the lactone ring of the drug. Data were collected through the IFC interface and analyzed on Chromaster software.

### Cell Proliferation Assay

Cells were seeded at a density of five thousand cells per well in 96-well plates and left to incubate overnight. Triplicate samples of free SN38, free SN38-G, and SN38-G liposomes were added at various dilutions and incubated for 24 h. CCK-8 stock solution was thawed at 37 °C in a water bath, 10 μL was added to each well, and plates were placed in the incubator for up to 4 h before measuring the absorbance at 450 nm using a microplate reader. Inhibition of cell proliferation was determined as

2 $$\prime \prime \%\;of\;inhibition\;compared\;to\;{control}^{\prime \prime }=absorbance\;at\;450\;nm\left(sample\right)\times 100/Absorbance\;at\;450\;nm\left(control\right)$$

### Cellular Regeneration of SN38

MCF-7 and MDA-MB-231 were seeded in triplicate at 10^5^ cells per well in 96-well plates and incubated overnight. SN38-G liposomes were added at 2 μM SN38-G in 100 μL of total medium to the cells with or without 100 μM beta-glucuronidase inhibitor, d-saccharic acid 1,4-lactone. The medium was harvested at 0, 6 h, 12 h, 24 h, and 48 h of incubation with the liposomes, and 100 μL of HPLC mobile phase supplemented with 1% Triton X-100 was added and incubated 10 min at 70 °C. 20 μL was injected in the analytical HPLC column and detected at 375/540 nm (ex/em).

### Statistical Analysis

Results are presented as the mean plus or minus (±) standard deviation (S.D.). All experiments were repeated multiple times, and representative data was shown. Statistical analyses were examined using the two-tailed unpaired Student’s *t*-test.

## Supplementary Material



## Notes

Additional experimental procedures, physicochemical characterization of the liposomes, drug partitioning studies, *in vitro* drug’s integrity assays, cytotoxicity evaluations in cancerous and non-cancerous cell lines, and a summary table of glucuronide prodrug designs and preclinical testing (PDF)
