# Supplementary material for: Safe and Efficient Intracellular Release of SN38 via Lysosomal-Responsive SN38‑G Loaded Liposomes
Source: ACS Appl Bio Mater. 2025 Jul 8;8(7):6231–42. doi: 10.1021/acsabm.5c00722 (PMC12284848; doi:10.1021/acsabm.5c00722)
Supplement: Supplementary file 1 [file mt5c00722_si_001.pdf]

Supporting Information

Safe and Efficient Intracellular Release of SN38 via Lysosomal-Responsive SN38-G Loaded Liposomes

Pierre-Alain Burnouf [1,], Yu-Cheng Su [2], Shih-Hung Yang [3]*

[1] International Center for Wound Repair and Regeneration, National Cheng-Kung University, Tainan, Taiwan.

[2] Department of Biological Science and Technology, National Yang Ming Chiao Tung University, Hsinchu, Taiwan.

[3] Institute of Biomedical Sciences, Academia Sinica, Taipei, Taiwan.

Corresponding Author:

Pierre-Alain Burnouf: International Center for Wound Repair and Regeneration, School of Medicine, National Cheng Kung University, Tainan City 701, Taiwan; orcid.org/0000-0002-6135-1547; Email: burnoufp@gs.ncku.edu.tw

Authors:

Yu-Cheng Su: Department of Biological Science and Technology, Center for Intelligent Drug Systems and Smart Bio-devices (IDS2B), National Yang Ming Chiao Tung University, Hsinchu 300, Taiwan; orcid.org/0000-0001-7056-6315

Shih-Hung Yang: Institute of Biomedical Sciences, Academia Sinica, Taipei 115, Taiwan; orcid.org/0009-0006-6003-9072

Supplementary Methods

Sequential Esterification, Hydrolysis, Enzymatic Digestion, and Biological Activity Assessment of SN38-G to SN38. Commercial SN38-G was esterified in methanol or ethanol using sulfuric acid (H_2SO_4) and DMSO, following the procedure detailed in the Methods section under Synthesis of amphiphilic SN38-G^{met} and SN38-G^{eth} from SN38-G. The formation of the esters was monitored by analytical HPLC. For analysis, 2 µL of the reaction mixture was diluted in 248 µL of HPLC mobile phase, and 2.5 µL was injected with the fluorescence detector set at an excitation/emission wavelength of 375/430 nm. Hydrolysis of SN38-G^{met} and SN38-G^{eth} was carried out at 70 °C in 10 mM HEPES buffer, adjusted to pH 8. Samples were taken at 0, 5, 30, and 60 minutes, diluted in HPLC mobile phase (70% methanol, 25 mM citric acid, pH 3), and incubated at 70 °C for 10 minutes before injection. To generate active SN38, SN38-G was enzymatically cleaved by beta-glucuronidase following pH adjustment to 7 with phosphate buffer. The biological activity of the resulting SN38 was evaluated in an in-vitro cytotoxicity assay using human MCF-7 breast cancer cells, in comparison to commercial SN38. Cells were seeded at 5,000 cells per well in 96-well plates and incubated overnight. Triplicate samples of either commercial SN38 or SN38 generated from SN38-G were added at various dilutions and incubated for 24 hours. Afterward, 10 µL of CCK-8 solution (pre-warmed to 37 °C) was added to each well, and the plates were returned to the incubator for up to 4 hours. Absorbance was then measured at 450 nm using a microplate reader. Cell proliferation inhibition was calculated as: % inhibition = [Abs_{450}(sample) / Abs_{450}(control)] × 100.

Cytotoxicity Assay in Cancerous and Non-Cancerous Cell Lines. Human breast adenocarcinoma MCF-7 cells and non-cancerous NIH/3T3 fibroblasts were cultured in DMEM (Dulbecco's Modified Eagle Medium) supplemented with 5% heat-inactivated FBS, 3.7 g/L sodium bicarbonate, 100 units/mL penicillin, and 100 µg/mL streptomycin. All cells were incubated at 37 °C in a humidified atmosphere with 5% CO_2. For the cytotoxicity assay cells were seeded at a density of 5,000 cells per well in 96-well plates and allowed to adhere overnight. SN38-G liposomes were prepared as described in the main Methods section and diluted in cell culture medium to obtain a series of decreasing concentrations. Cells were treated with the liposomal formulations for 24 hours. After treatment, 10 µL of CCK-8 reagent was added to each well and incubated up to 4 hours at 37 °C. Absorbance at 450 nm was measured using a microplate reader. Cell viability was calculated as a percentage of untreated control cells.

Distribution Between Soybean Oil and Water (Log D Oil-to-Water). The distribution between soybean oil and water was determined in 500 µL of 10 mM MES pH 6 and 500 µL of pure soybean oil after addition of 5 nmol of SN38 or SN38-G derivatives.. Equilibration between the 2 phases took place for 24 hours at

room temperature under constant shaking. After which, the tubes were centrifuged at 20,000 x g for 30 min at 24˚C, and samples from each phase were analyzed by analytical HPLC, using 70% methanol mobile phase. The final Log D (distribution coefficient) was determined by the Log of the fraction of the amount of drug found in the soybean oil phase over the amount of drug found in the water phase as follows:

Log D (oil-to-water) = Log ([drug]oil/[drug]water)

Comparative cytotoxicity including liposomal SN38 and CPT-11 against MCF-7 Cells. Cells were seeded at a density of five thousand cells per well in 96-well plates and incubated overnight. Liposomal SN38 formulations were purified by size-exclusion chromatography to remove unencapsulated drug and residual DMSO prior to dilution. Triplicate samples of liposomal SN38 and free CPT-11 were then added at various dilutions, ensuring that the final DMSO concentration in the cell culture medium was well below 0.1%, a level considered non-toxic for most cell types. Treatments were incubated for 24 hours for comparison against free SN38 and liposomal SN38-G. CCK-8 stock solution was thawed at 37˚C in a water bath, and 10 µL were added to each well. Plates were then incubated for up to 4 hours before measuring absorbance at 450 nm using a microplate reader.

Cytotoxicity Assay of Empty Liposomes. Human breast adenocarcinoma MCF-7 cells and non-cancerous NIH/3T3 fibroblasts were cultured in DMEM (Dulbecco's Modified Eagle Medium) supplemented with 5% heat-inactivated FBS, 3.7 g/L sodium bicarbonate, 100 units/mL penicillin, and 100 µg/mL streptomycin. Cells were maintained at 37 °C in a humidified atmosphere with 5% CO_2. For the cytotoxicity assay, cells were seeded in 96-well plates at a density of 5,000 cells per well and allowed to adhere overnight. Drug-free liposomes (containing calcium acetate but no SN38-G) were prepared as described in the main Methods section and diluted in culture medium to match the lipid concentrations equivalent to those used in the SN38-G liposome treatments. Cells were incubated with these empty liposomes for 24 hours. Following treatment, 10 µL of CCK-8 reagent was added to each well and incubated for up to 4 hours at 37 °C. Absorbance at 450 nm was measured using a microplate reader. Cell viability was expressed as a percentage relative to untreated control cells.

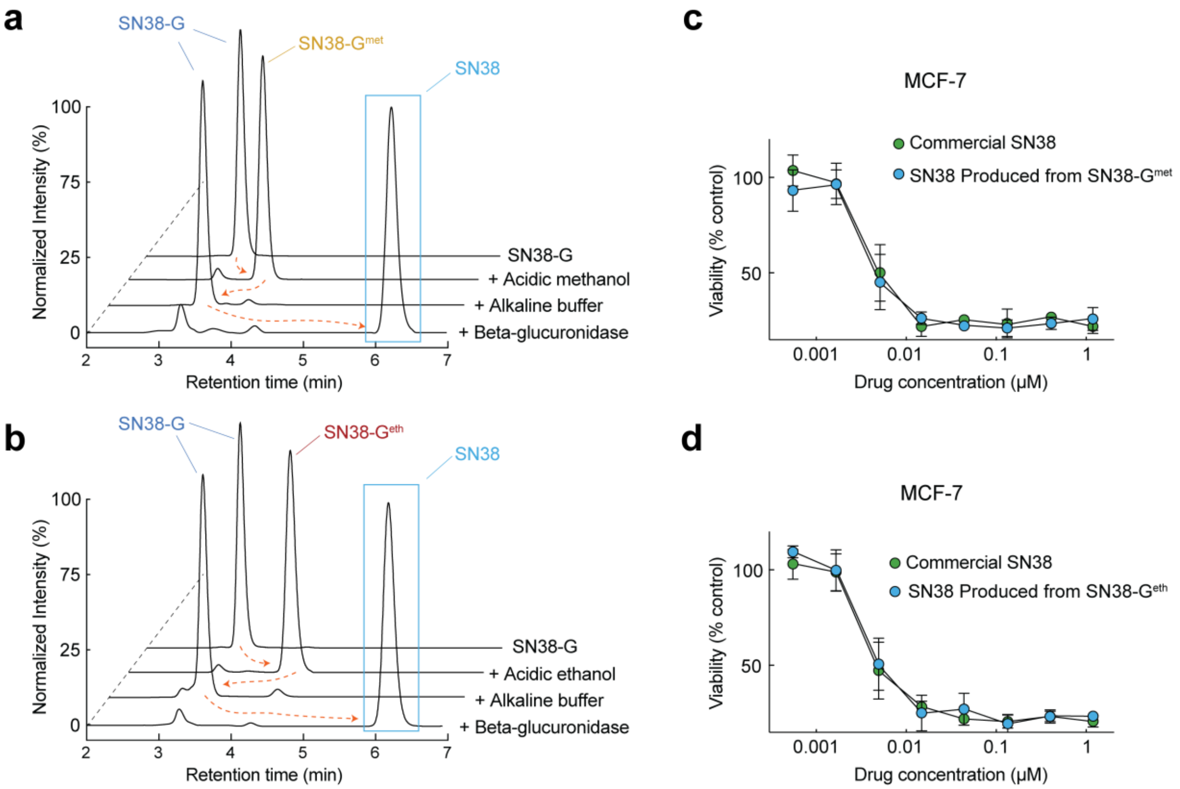

Supplementary Figure S1. Sequential esterification, hydrolysis, enzymatic digestion and biological activity of SN38-G to SN38. SN38-G was esterified in a mixture of methanol (a) or ethanol (b) and sulfuric acid (H_2SO_4) at elevated temperatures. The reverse reactions of hydrolysis were performed in 10 mM HEPES at pH 8. We observed complete de-esterification of SN38-G^{met} and SN38-G^{eth} back to SN38-G. SN38 was then formed from SN38-G by enzymatic digestion using beta-glucuronidase at 37˚C. (c, d) The biological activity of SN38 produced after esterification, hydrolysis and enzymatic digestion of SN38-G was compared with commercial SN38 against human breast cancer cells MCF-7. Error bars: SD, n = 3.

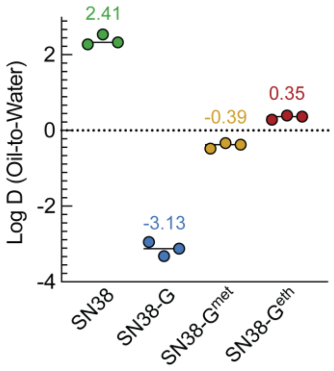

Supplementary Figure S2. Distribution of SN38 and Glucuronide Derivatives Between Soybean Oil and Water. Evaluation of the distribution behavior of SN38, SN38-G, SN38-G^{met}, and SN38-G^{eth} between soybean oil and an aqueous phase buffered at pH 6. $n = 3$

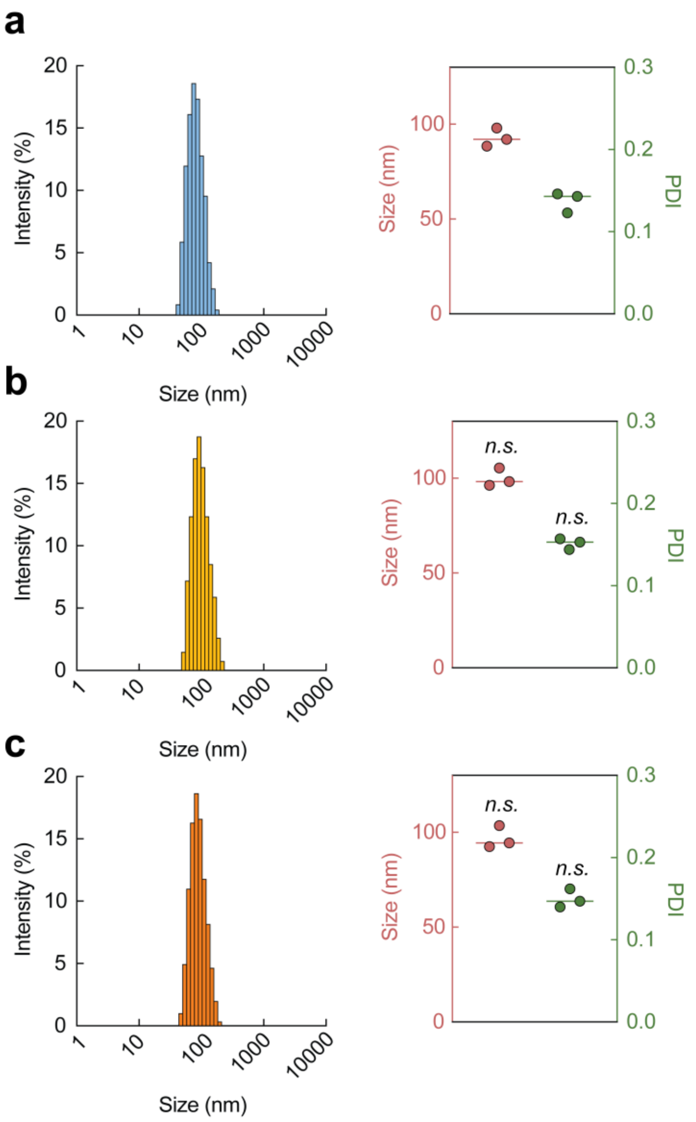

Supplementary Figure S3. Size and polydispersity index (PDI) of liposomes before and after drug loading. Dynamic light scattering (DLS) analysis of liposomes before loading (a), and after loading SN38-G^{met} (b), or SN38-G^{eth} (c). Measurements were performed in triplicate using a Malvern Zetasizer. No significant differences were observed in hydrodynamic diameter or PDI after drug loading compared to non-loaded liposomes. n = 3, statistical significance of differences in mean values: *n.s* not significant.

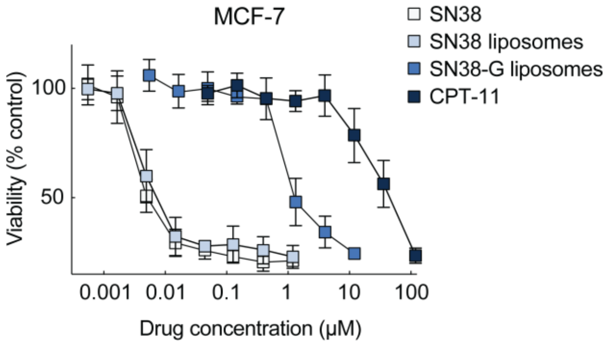

Supplementary Figure S4. Comparative cytotoxicity including liposomal SN38 and CPT-11 against MCF-7 Cells. Dose-dependent cytotoxic effects of liposomal SN38 and free CPT-11 on MCF-7 breast cancer cells after 24 hours of incubation. Cell viability was assessed using CCK8. Error bars: SD, $n = 3$.

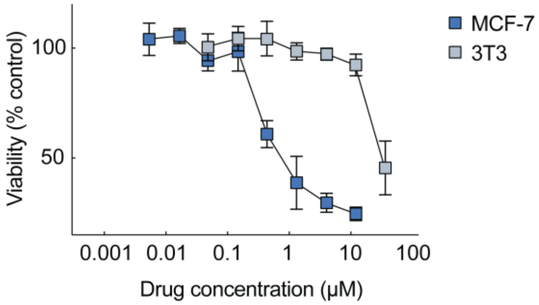

Supplementary Figure S5. Comparative cytotoxicity of SN38-G liposomes in cancerous and non-cancerous cell lines. Dose-dependent cytotoxic effects of SN38-G liposomes were assessed in MCF-7 human breast adenocarcinoma cells and non-cancerous NIH/3T3 fibroblasts. Cells were incubated with increasing concentrations of SN38-G liposomes for 24 hours, followed by evaluation of cell viability using the CCK-8 assay. Error bars: SD, n = 3.

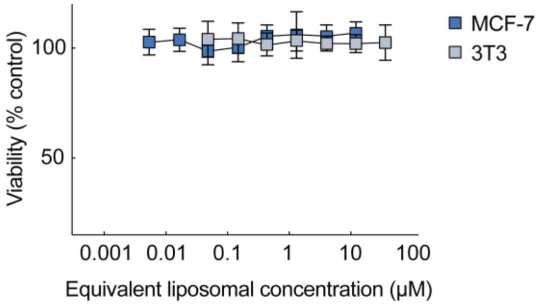

Supplementary Figure S6. Dose-dependent cytotoxic effects of drug-free liposomes were evaluated in MCF-7 human breast adenocarcinoma cells and non-cancerous NIH/3T3 fibroblasts. Cells were incubated with empty liposomes at lipid concentrations equivalent to those used in SN38-G liposome treatments for 24 hours. Cell viability was assessed using the CCK-8 assay. Error bars: SD, n = 3.

Supplementary Table S1. Design and preclinical testing of glucuronide-modified drugs.

Parental drug	Delivery	Preclinical parameters	Animal model	Preclinical outcome	Ref
**9-Amino Camptothecin (9AC)**	Free prodrug	In-vivo activity & Tolerance	BALB/c nu/nu & BALB/c mice	- Superior activity against lung adenocarcinoma (CL1-5) compared to colorectal adenocarcinoma (LS174T)<br>- Up to 85% of CL1-5-inoculated mice cured<br>- 9AC glucuronide was less toxic to older female mice at 50 mg/kg	[1]
	Free prodrug	In-vivo activity	BALB/c nu/nu & NOD/SCID mice	- Potent activity in BALB/c but not in NOD/SCID mice<br>- Infiltrating neutrophils and macrophages (absent from NOD/SCID mice) play a significant role in the activity	[2]
	Free prodrug & GDEPT	In-vivo activity	SCID-Beige mice	- Modified cells expressing surface mouse beta-glucuronidase show higher activity<br>- In GDEPT, 9AC glucuronide had lesser activity than p-hydroxy aniline (HAM) glucuronide	[3]
	Liposomes	In-vivo activity, mechanism, tolerance & pharmacokinetics	NOD/SCID mice	- Lysosomal beta-glucuronidase reformed 9AC after endocytosis of the liposomes<br>- 100% of mice cured from liposomal 9AC glucuronide at 10 mg/kg against breast adenocarcinoma (MDA-MB-468)<br>- Absence of toxicity<br>- 6 hours post-injection, the liposomal form increased the blood concentration of 9AC glucuronide 350-fold	[4]
**Cyclooctynes**	Micelles	In-vivo imaging	BALB/c athymic mice	- DiO is released from micelles at tumor sites after sequential enzymatic and bioorthogonal reactions	[5]
**Daunorubicin**	ADEPT	Pharmacokinetics & biodistribution	BALB/c & athymic nude (Harlan CPB) mice	- Half-life of human beta-glucuronidase antibody conjugate increased from 24 minutes to 9 hours after deglycosylation<br>- Accurate targeting of ovarian tumors (OVCAR-3) from the human beta-glucuronidase antibody conjugate<br>- The blood-to-tumor ratio of the conjugate was >2 7 days post-administration	[6]
**Doxorubicin**	Free prodrug	In-vivo activity & biodistribution	Athymic nude mice	- 5-fold lower heart accumulation and 5-fold higher tumor accumulation in ovarian cancer model (OVCAR-3) of doxorubicin glucuronide compared to doxorubicin<br>- 87% tumor inhibition of doxorubicin glucuronide against 56% for doxorubicin at an equitoxic dose<br>- Higher activity in large tumors	[7]
	Free prodrug	Biodistribution & pharmacokinetics	NMRI (Harlan) mice	- Methylesterification of doxorubicin glucuronide increase the tumor accumulation 2.7-fold in an ovarian cancer model (FMa)<br>- Methylesterification slows down clearance from blood	[8]
	Free prodrug	Biodistribution, tolerance & in-vivo activity	NMRI nu/nu, MF-1, CD-1 nu/nu mice & monkeys	- Diminished heart accumulation & 10-fold higher tumor accumulation in a subcutaneous human carcinoma xenograft<br>- LD10 was 307.3 mg/kg in male mice and 623.9 mg/kg in female mice	[9]

				- No apparent toxicity in monkeys at 250 mg/kg apart from a transient leukocyte count reduction<br>- Superior activity compared to doxorubicin with growth inhibition and complete remission in carcinomas from human lung, colon, breast, ovary, and stomach	
	ADEPT	Biodistribution, pharmacokinetics & in-vivo activity	CD-1 nu/nu mice, CD rats & monkeys	- Amounts of drug were 4 - 12-fold higher after fusion protein treatment in a stomach carcinoma model (Mz-Sto-1)<br>- Mice and rats had higher blood concentration than monkeys at 50 mg/kg post i.v. injection<br>- Significant growth delay and partial regression were observed with doxorubicin glucuronide only for mice treated with the fusion protein	10
	ADEPT	In-vivo activity and tolerance	Athymic nude mice	- Doxorubicin glucuronide had a maximum tolerated dose of 500 mg/kg (twice weekly) against only 8 mg/kg (twice weekly) for doxorubicin<br>- Ovarian tumor (FMa) inhibition was superior when mice were treated with the fusion protein	11
**p-hydroxy aniline (HAM)**	Free prodrug & GDEPT	In-vivo activity	SCID-Beige, BALB/c mice	- 100% regression in mice bearing bladder carcinoma tumors (EJ) expressing surface mouse beta-glucuronidase<br>- Increased activity against colon carcinoma (CT26) tumors expressing surface mouse beta-glucuronidase	3
	ADEPT	In-vivo activity & bystander effect	BALB/c nu/nu	- Observed bystander effect in mixed tumors of antigen-expressing and non-expressing cells of rat hepatoma (AS-30D & N1S1)<br>- 100% of mice were cured in antigen-expressing group	12
	ADEPT	In-vivo activity, biodistribution & pharmacokinetics	BALB/c nu/nu & SCID-Beige mice	- Fusion protein had a terminal half-life of 1.9h and accumulated in colon adenocarcinoma (LS174T) tumors and liver<br>- The fusion protein was less active in the liver, maybe due to internalization by local cells<br>- Significant tumor growth inhibition and partial regression	13
**Poly (ADP-ribose) polymerase inhibitor (TSL-1502)**	Free prodrug	In-vivo activity, toxicity & biodistribution	BALB/c nude mice	- No body weight loss and complete breast adenocarcinoma (MDA-MB-436) tumor regression at 50 mg/kg<br>- 91% inhibition at 50 mg/kg, and 2 out of 6 mice had partial tumor regression against mammary carcinoma (MX-1) tumors<br>- Combination with carboplatin led to complete tumor regression with a slight body weight loss of 3.8%<br>- In a colon adenocarcinoma model (SW620), effective tumor inhibition was observed at 5 mg/kg when combined with CPT-11 without weight loss	14
**Monomethylauristatin E (MMAE)**	Free prodrug	In-vivo activity	C57BL/6 mice	- MMAE glucuronide was as effective as doxorubicin glucuronide at a 237-fold lower dose against lung carcinoma (LLC) tumors	15

	ADC	In-vivo activity & tolerance	SCID mice	- 100% cure against non-Hodgkin's lymphoma (Karpas 299) at a single 0.5 mg/kg dose<br>- Well tolerated up to 100 mg/kg dosage	[16]
	ADC	In-vivo activity & pharmacokinetics	SCID mice & Harlan SD rats	- The introduction of the side chain PEG24 in the ADC design significantly enhanced its activity against the Hodgkin's lymphoma (L540cy) model<br>- The side chain improved the pharmacokinetics of an ADC design with a DAR of 8	[17]
	ADC	In-vivo activity, tolerance & pharmacokinetics	SCID, BALB/c mice & SD rats	- DAR 8 / PEG_8 side chain design cured 5 out of 5 mice at a single 3 mg/kg dose against $CD19^+$ large B-cell lymphoma (DLBCL)<br>- DAR 8 / PEG_{12} side chain design cured 10 out of 10 mice at a single 3 mg/kg dose against $CD19^+$ Burkitt's lymphoma (Raji)<br>- DAR 8 / PEG_{12} side chain design showed complete regression in 10 out of 10 mice at a single 3 mg/kg dose against $CD19^+$ WSU-DLBCL<br>- DAR 8 / $PEG_{>8}$ side chain design ADCs were well tolerated at 50 mg/kg but not at $PEG_{<8}$ side chain<br>- DAR 8 / $PEG_{>8}$ side chain displayed similar pharmacokinetics to the parental antibody	[18]
	ADC	In-vivo activity & tolerance	CB17/SCID, NOD/SCID mice & SD rats	- Tandem-cleavable linker ADC construct was effective at 3 mg/kg and a single dose against mantle cell lymphoma (JeKo-1)<br>- 6 out of 6 mice had complete response at a single injection of 10 mg/kg against B cell lymphoma (Granta 519)<br>- Well tolerated at 40 mg/kg without evidence of myelosuppression	[19]
	ADC	In-vivo activity & pharmacokinetics	SCID mice & Sprague-Dawley rats	- Polysarcosine had superior activity compared to the PEG ADC construct at equal length against breast carcinoma (BT-474)<br>- At 12 repeated units, polysarcosine had similar pharmacokinetics properties as the parental antibody	[20]
	Albumine conjugate	Tolerance & In-vivo activity	BALB/c athymic, & nude mice	- Well tolerated at 8 mg/kg<br>- Albumin binding improved the efficacy of MMAE glucuronide against mouth epidermal carcinoma (KB)<br>- Significant tumor volume reduction against orthotopic TNBC tumors (MDA-MB-231) at 4 mg/kg weekly for 5 week<br>- 33% tumor-free against orthotopic pancreatic carcinoma (MIA PaCa2) at 4 mg/kg (twice)<br>- Effective against large tumors (> 3000 nm^3)<br>- 4 mg/kg caused no body weight loss	[21]
	Albumine conjugate	In-vivo activity & tolerance	BALB/c athymic mice	- MMAE released through dimeric linkage had similar in-vivo activity as its monomeric analog against colorectal adenocarcinoma (LS174T)<br>- Absence of observable side-effects	[22]

	Albumine conjugate	In-vivo activity & tolerance	BALB/c nude mice	- Tumor remission in 3 out of 8 mice against pancreatic carcinoma (MIA PaCa2)<br>- No body weight loss	23
	Monotherapy, albumin conjugate / nanoparticles	In-vivo activity & pharmacokinetics	BALB/c-nu mice	- Well tolerated but absence of in-vivo activity against breast adenocarcinoma (MDA-MB-231) as monotherapy treatment<br>- Significant anticancer activity in a construct including a trityl group PEG albumin binder supramolecular design<br>- Albumin binding was necessary to provide in-vivo activity	24
**Monomethylauristatin F (MMAF)**	ADC	In-vivo activity & tolerance	SCID mice	- 2 out of 7 mice cured against renal cell carcinoma (RCC) at 0.75 mg/kg<br>- Well tolerated at 25 mg/kg	16
**Phenolphthalein $^{131}I/^{124}I$**	Probe-based	Imaging	BALB/c mice	- Glucuronidation of the probe helps the imaging of beta-glucuronidase expressing colon carcinoma (CT26) tumors	25
**Pyrrolobenzodiazepine dimer (PBD)**	ADC	In-vivo activity & tolerance	SCID mice	- Growth delays at sub-curative dosage of 1 mg/kg against low HER2 breast carcinoma (JIMT-1) and high HER2 gastric carcinoma (NCI-N87)<br>- In high HER2 tumors, a sustained tumor regression was observed with 6 complete responses and 4 partial responses at 3 mg/kg<br>- Absence of body weight loss	26
**Tubulysin**	ADC	In-vivo activity & bystander effect	SCID mice	- Potent against MDR+ tumors together with bystander effect in a heterogenous antigen expression model<br>- Protection against acetate hydrolysis of linkers for improved ADC in-vivo activity	27, 28

References

(1) Prijovich, Z. M.; Chen, B. M.; Leu, Y. L.; Chern, J. W.; Roffler, S. R. Anti-tumour activity and toxicity of the new prodrug 9-aminocamptothecin glucuronide (9ACG) in mice. *Br J Cancer* **2002**, *86* (10), 1634-1638. DOI: 10.1038/sj.bjc.6600317.
(2) Juan, T. Y.; Roffler, S. R.; Hou, H. S.; Huang, S. M.; Chen, K. C.; Leu, Y. L.; Prijovich, Z. M.; Yu, C. P.; Wu, C. C.; Sun, G. H.; et al. Antiangiogenesis targeting tumor microenvironment synergizes glucuronide prodrug antitumor activity. *Clin Cancer Res* **2009**, *15* (14), 4600-4611. DOI: 10.1158/1078-0432.CCR-09-0090.
(3) Chen, K. C.; Cheng, T. L.; Leu, Y. L.; Prijovich, Z. M.; Chuang, C. H.; Chen, B. M.; Roffler, S. R. Membrane-localized activation of glucuronide prodrugs by beta-glucuronidase enzymes. *Cancer Gene Ther* **2007**, *14* (2), 187-200. DOI: 10.1038/sj.cgt.7700999.
(4) Burnouf, P. A.; Leu, Y. L.; Su, Y. C.; Wu, K.; Lin, W. C.; Roffler, S. R. Reversible glycosidic switch for secure delivery of molecular nanocargos. *Nat Commun* **2018**, *9* (1), 1843. DOI: 10.1038/s41467-018-04225-5.
(5) Porte, K.; Renoux, B.; Peraudeau, E.; Clarhaut, J.; Eddhif, B.; Poinot, P.; Gravel, E.; Doris, E.; Wijkhuisen, A.; Audisio, D.; et al. Controlled Release of a Micelle Payload via Sequential Enzymatic and Bioorthogonal Reactions in Living Systems. *Angew Chem Int Ed Engl* **2019**, *58* (19), 6366-6370. DOI: 10.1002/anie.201902137.
(6) Houba, P. H.; Boven, E.; Haisma, H. J. Improved characteristics of a human beta-glucuronidase-antibody conjugate after deglycosylation for use in antibody-directed enzyme prodrug therapy. *Bioconjug Chem* **1996**, *7* (5), 606-611. DOI: 10.1021/bc960055j.
(7) Houba, P. H.; Boven, E.; van der Meulen-Muileman, I. H.; Leenders, R. G.; Scheeren, J. W.; Pinedo, H. M.; Haisma, H. J. A novel doxorubicin-glucuronide prodrug DOX-GA3 for tumour-selective chemotherapy: distribution and efficacy in experimental human ovarian cancer. *Br J Cancer* **2001**, *84* (4), 550-557. DOI: 10.1054/bjoc.2000.1640.
(8) de Graaf, M.; Nevalainen, T. J.; Scheeren, H. W.; Pinedo, H. M.; Haisma, H. J.; Boven, E. A methylester of the glucuronide prodrug DOX-GA3 for improvement of tumor-selective chemotherapy. *Biochem Pharmacol* **2004**, *68* (11), 2273-2281. DOI: 10.1016/j.bcp.2004.08.004.
(9) Bosslet, K.; Straub, R.; Blumrich, M.; Czech, J.; Gerken, M.; Sperker, B.; Kroemer, H. K.; Gesson, J. P.; Koch, M.; Monneret, C. Elucidation of the mechanism enabling tumor selective prodrug monotherapy. *Cancer Res* **1998**, *58* (6), 1195-1201.
(10) Bosslet, K.; Czech, J.; Hoffmann, D. Tumor-selective prodrug activation by fusion protein-mediated catalysis. *Cancer Res* **1994**, *54* (8), 2151-2159.
(11) Houba, P. H.; Boven, E.; van der Meulen-Muileman, I. H.; Leenders, R. G.; Scheeren, J. W.; Pinedo, H. M.; Haisma, H. J. Pronounced antitumor efficacy of doxorubicin when given as the prodrug DOX-GA3 in combination with a monoclonal antibody beta-glucuronidase conjugate. *Int J Cancer* **2001**, *91* (4), 550-554. DOI: 10.1002/1097-0215(200002)9999:9999<::aid-ijc1075>3.0.co;2-l.
(12) Cheng, T. L.; Wei, S. L.; Chen, B. M.; Chern, J. W.; Wu, M. F.; Liu, P. W.; Roffler, S. R. Bystander killing of tumour cells by antibody-targeted enzymatic activation of a glucuronide prodrug. *Br J Cancer* **1999**, *79* (9-10), 1378-1385. DOI: 10.1038/sj.bjc.6690221.

(13) Chen, K. C.; Wu, S. Y.; Leu, Y. L.; Prijovich, Z. M.; Chen, B. M.; Wang, H. E.; Cheng, T. L.; Roffler, S. R. A humanized immunoenzyme with enhanced activity for glucuronide prodrug activation in the tumor microenvironment. *Bioconjug Chem* **2011**, *22* (5), 938-948. DOI: 10.1021/bc1005784.
(14) Wang, L.; Zhu, X.; Li, L.; Li, L.; Fu, L.; Li, Y.; Fu, H.; Chen, X.; Lou, L. TSL-1502, a glucuronide prodrug of a poly (ADP-ribose) polymerase (PARP) inhibitor, exhibits potent anti-tumor activity in preclinical models. *Am J Cancer Res* **2021**, *11* (4), 1632-1645.
(15) Legigan, T.; Clarhaut, J.; Renoux, B.; Tranoy-Opalinski, I.; Monvoisin, A.; Jayle, C.; Alsarraf, J.; Thomas, M.; Papot, S. Synthesis and biological evaluations of a monomethylauristatin E glucuronide prodrug for selective cancer chemotherapy. *Eur J Med Chem* **2013**, *67*, 75-80. DOI: 10.1016/j.ejmech.2013.06.037.
(16) Jeffrey, S. C.; Andreyka, J. B.; Bernhardt, S. X.; Kissler, K. M.; Kline, T.; Lenox, J. S.; Moser, R. F.; Nguyen, M. T.; Okeley, N. M.; Stone, I. J.; et al. Development and properties of beta-glucuronide linkers for monoclonal antibody-drug conjugates. *Bioconjug Chem* **2006**, *17* (3), 831-840. DOI: 10.1021/bc0600214.
(17) Lyon, R. P.; Bovee, T. D.; Doronina, S. O.; Burke, P. J.; Hunter, J. H.; Neff-LaFord, H. D.; Jonas, M.; Anderson, M. E.; Setter, J. R.; Senter, P. D. Reducing hydrophobicity of homogeneous antibody-drug conjugates improves pharmacokinetics and therapeutic index. *Nat Biotechnol* **2015**, *33* (7), 733-735. DOI: 10.1038/nbt.3212.
(18) Burke, P. J.; Hamilton, J. Z.; Jeffrey, S. C.; Hunter, J. H.; Doronina, S. O.; Okeley, N. M.; Miyamoto, J. B.; Anderson, M. E.; Stone, I. J.; Ulrich, M. L.; et al. Optimization of a PEGylated Glucuronide-Monomethylauristatin E Linker for Antibody-Drug Conjugates. *Mol Cancer Ther* **2017**, *16* (1), 116-123. DOI: 10.1158/1535-7163.MCT-16-0343.
(19) Chuprakov, S.; Ogunkoya, A. O.; Barfield, R. M.; Bauzon, M.; Hickle, C.; Kim, Y. C.; Yeo, D.; Zhang, F.; Rabuka, D.; Drake, P. M. Tandem-Cleavage Linkers Improve the In Vivo Stability and Tolerability of Antibody-Drug Conjugates. *Bioconjug Chem* **2021**, *32* (4), 746-754. DOI: 10.1021/acs.bioconjchem.1c00029.
(20) Viricel, W.; Fournet, G.; Beaumel, S.; Perrial, E.; Papot, S.; Dumontet, C.; Joseph, B. Monodisperse polysarcosine-based highly-loaded antibody-drug conjugates. *Chem Sci* **2019**, *10* (14), 4048-4053. DOI: 10.1039/c9sc00285e.
(21) Renoux, B.; Raes, F.; Legigan, T.; Peraudeau, E.; Eddhif, B.; Poinot, P.; Tranoy-Opalinski, I.; Alsarraf, J.; Koniev, O.; Kolodych, S.; et al. Targeting the tumour microenvironment with an enzyme-responsive drug delivery system for the efficient therapy of breast and pancreatic cancers. *Chem Sci* **2017**, *8* (5), 3427-3433. DOI: 10.1039/c7sc00472a.
(22) Renoux, B.; Fangous, L.; Hotten, C.; Peraudeau, E.; Eddhif, B.; Poinot, P.; Clarhaut, J.; Papot, S. A beta-glucuronidase-responsive albumin-binding prodrug programmed for the double release of monomethyl auristatin E. *Medchemcomm* **2018**, *9* (12), 2068-2071. DOI: 10.1039/c8md00466h.
(23) Chatre, R.; Lange, J.; Peraudeau, E.; Poinot, P.; Lerondel, S.; Le Pape, A.; Clarhaut, J.; Renoux, B.; Papot, S. In vivo synthesis of triple-loaded albumin conjugate for efficient targeted cancer chemotherapy. *J Control Release* **2020**, *327*, 19-25. DOI: 10.1016/j.jconrel.2020.08.008.

(24) Jarlstad Olesen, M. T.; Walther, R.; Poier, P. P.; Dagnaes-Hansen, F.; Zelikin, A. N. Molecular, Macromolecular, and Supramolecular Glucuronide Prodrugs: Lead Identified for Anticancer Prodrug Monotherapy. *Angew Chem Int Ed Engl* **2020**, *59* (19), 7390-7396. DOI: 10.1002/anie.201916124.
(25) Tzou, S. C.; Roffler, S.; Chuang, K. H.; Yeh, H. P.; Kao, C. H.; Su, Y. C.; Cheng, C. M.; Tseng, W. L.; Shiea, J.; Harm, I. H.; et al. Micro-PET imaging of beta-glucuronidase activity by the hydrophobic conversion of a glucuronide probe. *Radiology* **2009**, *252* (3), 754-762. DOI: 10.1148/radiol.2523082055.
(26) Gregson, S. J.; Barrett, A. M.; Patel, N. V.; Kang, G. D.; Schiavone, D.; Sult, E.; Barry, C. S.; Vijayakrishnan, B.; Adams, L. R.; Masterson, L. A.; et al. Synthesis and evaluation of pyrrolobenzodiazepine dimer antibody-drug conjugates with dual beta-glucuronide and dipeptide triggers. *Eur J Med Chem* **2019**, *179*, 591-607. DOI: 10.1016/j.ejmech.2019.06.044.
(27) Burke, P. J.; Hamilton, J. Z.; Pires, T. A.; Lai, H. W. H.; Leiske, C. I.; Emmerton, K. K.; Waight, A. B.; Senter, P. D.; Lyon, R. P.; Jeffrey, S. C. Glucuronide-Linked Antibody-Tubulysin Conjugates Display Activity in MDR(+) and Heterogeneous Tumor Models. *Mol Cancer Ther* **2018**, *17* (8), 1752-1760. DOI: 10.1158/1535-7163.MCT-18-0073.
(28) Hamilton, J. Z.; Pires, T. A.; Mitchell, J. A.; Cochran, J. H.; Emmerton, K. K.; Zaval, M.; Stone, I. J.; Anderson, M. E.; Jin, S.; Waight, A. B.; et al. Improving Antibody-Tubulysin Conjugates through Linker Chemistry and Site-Specific Conjugation. *ChemMedChem* **2021**, *16* (7), 1077-1081. DOI: 10.1002/cmdc.202000889.
